# *Plasmodium vivax* spleen-dependent protein 1 and its role in extracellular vesicles-mediated intrasplenic infections

**DOI:** 10.3389/fcimb.2024.1408451

**Published:** 2024-05-17

**Authors:** Alberto Ayllon-Hermida, Marc Nicolau-Fernandez, Ane M. Larrinaga, Iris Aparici-Herraiz, Elisabet Tintó-Font, Oriol Llorà-Batlle, Agnes Orban, María Fernanda Yasnot, Mariona Graupera, Manel Esteller, Jean Popovici, Alfred Cortés, Hernando A. del Portillo, Carmen Fernandez-Becerra

**Affiliations:** ^1^ISGlobal, Barcelona Institute for Global Health, Hospital Clínic-Universitat de Barcelona, Barcelona, Spain; ^2^IGTP Institut d’Investigació Germans Trias i Pujol, Ctra. de Can Ruti, Barcelona, Spain; ^3^School of Medicine and Health Sciences, University of Barcelona, Barcelona, Spain; ^4^Endothelial Pathobiology and Microenvironment Group, Josep Carreras Leukaemia Research Institute (IJC), Barcelona, Catalonia, Spain; ^5^Malaria Research Unit, Institut Pasteur du Cambodge, Phnom Penh, Cambodia; ^6^Grupo de Investigaciones Microbiológicas y Biomédicas de Córdoba-GIMBIC, Universidad de Córdoba, Monteria, Colombia; ^7^ICREA, Institució Catalana de Recerca i Estudis Avançats, Barcelona, Spain; ^8^CIBERONC, Centro de Investigacion Biomedica en Red Cancer, Instituto de Salud Carlos III, Madrid, Spain; ^9^Cancer Epigenetics Group, Josep Carreras Leukaemia Research Institute (IJC), Barcelona, Catalonia, Spain; ^10^Physiological Sciences Department, School of Medicine and Health Sciences, University of Barcelona (UB), Barcelona, Catalonia, Spain; ^11^G5 Épidémiologie et Analyse des Maladies Infectieuses, Département de Santé Globale, Institut Pasteur, Paris, France; ^12^CIBERINFEC, ISCIII-CIBER de Enfermedades Infecciosas, Instituto de Salud Carlos III, Madrid, Spain

**Keywords:** *Plasmodium vivax*, intrasplenic infections, extracellular vesicles (EVs), CRISPR/Ca9, single-cell RNASeq (scRNASeq), spleen fibroblasts

## Abstract

Recent studies indicate that human spleen contains over 95% of the total parasite biomass during chronic asymptomatic infections caused by *Plasmodium vivax*. Previous studies have demonstrated that extracellular vesicles (EVs) secreted from infected reticulocytes facilitate binding to human spleen fibroblasts (hSFs) and identified parasite genes whose expression was dependent on an intact spleen. Here, we characterize the *P. vivax* spleen-dependent hypothetical gene (PVX_114580). Using CRISPR/Cas9, PVX_114580 was integrated into *P. falciparum* 3D7 genome and expressed during asexual stages. Immunofluorescence analysis demonstrated that the protein, which we named *P. vivax Spleen-Dependent Protein 1* (PvSDP1), was located at the surface of infected red blood cells in the transgenic line and this localization was later confirmed in natural infections. Plasma-derived EVs from *P. vivax*-infected individuals (PvEVs) significantly increased cytoadherence of 3D7_PvSDP1 transgenic line to hSFs and this binding was inhibited by anti-PvSDP1 antibodies. Single-cell RNAseq of PvEVs-treated hSFs revealed increased expression of adhesion-related genes. These findings demonstrate the importance of parasite spleen-dependent genes and EVs from natural infections in the formation of intrasplenic niches in *P. vivax*, a major challenge for malaria elimination.

## Introduction

Malaria caused by *Plasmodium vivax* infection (vivax malaria) is a global health issue. *P. vivax* is the most widely distributed human malaria parasite, ranging from South-East Asia to the Americas region, in an area where 2.5 billion people live at risk of transmission (Howes et al., 2016). Almost 7.7 million clinical cases were estimated in 2022 (World Health Organization, 2023). However, current available diagnostic methods only detect a small percentage of infection, as epidemiological field studies estimate that up to 90% of chronic infections are asymptomatic (White et al., 2018) and 50-80% are below the sensitivity of current diagnostics methods (Okell et al., 2012). Moreover, the degree of these infections varies across different endemic regions (Angrisano and Robinson, 2022). A better understanding of these asymptomatic infections is needed if elimination of malaria is to be achieved.

For many years, it was amply accepted that the dormant form of the liver, called hypnozoites (Krotoski et al., 1982; Krotoski, 1985), was the solely responsible for these asymptomatic infections (White, 2011). However, the reticulocyte-rich spleen has recently emerged as a major cryptic niche (Siqueira et al., 2012) where more than 95% of the total parasite biomass has been observed in natural infections (Kho et al., 2021b, 2021a). The reason for this tropism remains unclear, yet it seems a major cause of anemia (Kho et al., 2024). Of interest, parasite genes whose expression is dependent on an intact spleen, have been identified in experimental infections using a nonhuman primate model susceptible to *P. vivax* (Fernandez-Becerra et al., 2020). Moreover, this study suggested that infected reticulocytes expressing spleen-dependent proteins may adhere to the spleen’s microvasculature, specifically to fibroblasts expressing ICAM-1.

Extracellular vesicles (EVs) are double membrane particles secreted from cells and have recently emerged as relevant mediators of intercellular communication (Yáñez-Mó et al., 2015). EVs have been classified into two main categories, exosomes and microvesicles, based on their size, biogenesis, and composition (Raposo and Stoorvogel, 2013). Exosomes are 30-100 nm vesicles of endocytic origin that are released after the fusion of multivesicular bodies (MVBs) with the plasma membrane. Microvesicles are larger in size (0.2-2 um) and originate by budding and shedding from the plasma membrane. Exosomes were firstly described in reticulocytes (Harding et al., 1983; Pan and Johnstone, 1983), the host cell for *P. vivax* invasion in its life cycle. Of note, during their maturation to erythrocytes, reticulocytes selectively remove proteins through the formation of reticulocyte-derived exosomes. Noticeably, it was previously shown that EVs isolated from *P. vivax* individuals can increase the binding capacity to human spleen fibroblasts (hSF) of *P. vivax*-infected reticulocytes *in vitro* via NF-κB nuclear translocation (Toda et al., 2020).

CRISPR/Cas9 editing has accelerated dramatically our ability to test essential metabolic pathways, to conditionally express genes and to generate transgenic parasites which altogether have enabled us to gain better understanding of malaria parasites (Ghorbal et al., 2014; Adjalley and Lee, 2022). Due to the lack of *in vitro* culture, we used the CRISPR/Cas9 technology to generate a *P. falciparum* 3D7 transgenic line expressing a hypothetical *P. vivax* spleen-dependent gene (PVX_114580) (Fernandez-Becerra et al., 2020), from here on named as *P. vivax* spleen dependent protein 1 (PvSDP1). To functionally characterize it, we have performed binding assays of the transgenic line (3D7_PvSDP1) to hSFs previously stimulated with plasma-derived EVs from *P. vivax* patients (PvEVs). Moreover, we have demonstrated that this binding is partially inhibited when 3D7_PvSDP1 parasites are pre-treated with a polyclonal antibody against PvSDP1 generated in house. Of note, this antibody was also used to show that PvSDP1 is expressed at the surface of infected reticulocytes in *P. vivax* natural infections. Last, we performed single-cell RNAseq of hSFs stimulated with PvEVs from natural infections to get further insights into the formation of parasite intrasplenic niches.

## Material and methods

### Human plasma samples

Plasma from *P. vivax* patients used in this study was collected at the E.S.E. Hospital San José de Tierralta, Colombia. Signed informed consent was obtained from all patients by the Universidad de Córdoba, Monteria (Colombia). Samples from healthy donors were collected at the Hospital Germans Tries I Pujol, Badalona (Spain), with expressed consent from the donors. Information regarding patients’ parasitaemia, gender, age and other relevant information can be found in **Table EV1**. Ten milliliters of peripheral blood were collected in sodium citrate tubes, followed by centrifugation at 400 x g for 10 min at room temperature (RT). The collected plasmas underwent further centrifugation at 2000 x g for 10 min. The resulting plasma supernatant was recovered, aliquoted into 1 ml fractions, and subsequently frozen at -80°C.

### Plasmid constructs, parasite culture and transfection

Plasmid pHH1_DiCre_Lisp1, previously described (Llorà-Batlle et al., 2020), underwent modification to incorporate the Chloroquine Resistance Transporter Promoter (CRT Promoter) and a triple hemagglutinin tag (3HA). Initially, the CRT Promoter was excised from the pARL_Vir14 plasmid (Bernabeu et al., 2012) utilizing BglII-PstI restriction enzymes and subsequently ligated into the pBlueScript II KS (+) vector at the BamHI/PstI sites. This construct was then subcloned into the SpeI/PstI sites of the pHH1_DiCre_LISP1 plasmid. The 3HA tag was synthesized through the design and annealing of two complementary primers, F-HA: GGTACCATGCATACTAGTCCCGGGTACCCATACGACGTCCCAGACTACGCTTACCCATACGACGTCCCAGACTACGCT and R-HA: CCATACGACGTCCCAGACTACGCTTACCCATACGACGTCCCAGACTACGCTTACCCATACGACGTCCCAGACTACGCTTAATAACTGCAG and cloned into the modified pHH1_DiCre_Lisp1 at KpnI/PstI sites. A KpnI cloning site was deliberately preserved between the promoter sequence and the 3HA.

The *P. vivax* PVX_114580 gene was amplified from a cDNA template obtained from total RNA of the Sal-I strain (kind gift of Dr. John Barnwell) using primers F-PvSDP1: ACTCGACCCGGGATGGTACCATGAAGAGCATTTTGGGCC and R-PvSDP1: TGGGACGTCGTATGGGTACCAAACTTCTTTTGCTTATTTTTCTTT and Platinum™ *Taq* (Invitrogen 11304011). PVX_114580 was cloned at the KpnI restriction site using In-Fusion^®^ HD Cloning Plus CE (Takara 638916), following manufacturer´s instructions. The generated plasmid, pHH1_CRT-PvSDP1-3HA_LISP1, was cloned into SURE2 Competent Cells (Agilent 200152) and purified using EndoFree Plasmid Maxi Kit (Qiagen 12362).

The *P. falciparum* 3D7 strain was obtained from MR4 BEI Resources (MRA-102) and cultured with B+ human erythrocytes (3% hematocrit) in RPMI media (Sigma 51800-035) supplemented with 0.5% Albumax, 0.23% NaHCO_3_, 0.59% HEPES, 0.05% gentamicin and 0.05 mg/ml of hypoxanthine using standard methods (Trager and Jensen, 1976).

*P. falciparum* 3D7 WT parasites were transfected as described previously (Llorà-Batlle et al., 2020). Briefly, 60 μg of pDC2_Cas9_hDHFRyFCU1_LISP1 and 12 μg of pHH1_CRT-PvSDP1-3HA_LISP1 linearized using BsaI site (located in the ampicillin resistance cassette at the backbone of the plasmid) were used for transfection. Seven ml of 8% ring stage-synchronized culture were electroporated (310 V, 950 µF and 2 mm cuvette) with the above-mentioned plasmids. 24 h after electroporation, 10 nM WR99210 was added to the culture and maintained for 4 days. After assessing genomic integration, subcloning by limiting dilution was performed to obtain clonal population of transgenic parasites expressing PvSDP1.

### Genomic integration and transcript detection

Genomic DNA from the *P. falciparum* 3D7_PvSDP1 transgenic line was obtained from 10 ml cultures with 10% mature stages parasitaemia by using QIAamp^®^ DNA Blood Mini Kit (Qiagen 51104). Integration was confirmed by PCR using KAPA2G Robust HotStart ReadyMix (Sigma KK5701) with primers used for amplification of PVX_114580, as well as primers targeting the *lisp1* gene.

Total RNA was obtained from 16% mature-stages *P. falciparum* 3D7_PvSDP1 pellet after saponin lysis. Parasites were resuspended in TRIzol™ (Invitrogen 15596026) and incubated for 5 min at RT. Purification of RNA was performed using phenol:chloroform with precipitation in isopropanol. To minimize the risk of DNA contamination, RNA was DNAse treated (Invitrogen 8170G). First-strand cDNA synthesis was performed using 1 μg of total RNA quantified via 2200 TapeStation system (Agilent), and the SuperScript™ IV (Invitrogen 18091050) with random hexamers following manufacturer’s instructions. For RT-PCR, specific forward primer for PVX_114580 and reverse 3HA Tag primer were used using KAPA2G Robust HotStart ReadyMix.

### Western blotting

Trophozoite/schizont parasites were harvested at 15% parasitaemia using LS-MACS separation columns (Miltenyi 130-042-401). Briefly, 12 ml of *P. falciparum* 3D7_PvSDP1 culture was pelleted by centrifugation at 300 x g for 5 min. Cell pellet was resuspended in 4 ml of RPMI and loaded on top of a LS-MACS column previously equilibrated with RPMI. Then, the parasite preparation was allowed to enter the column while being placed in LS-MACS magnet separator. Afterwards, two washes of 8 ml each were applied to the column to remove ring-stage parasites. Finally, 4 ml of RPMI were added to the column and parasites were eluted by removing the column from the magnet and by using the plunger. Parasites were lysed using parasite lysis buffer (PBS, 4% SDS, 0.1% Triton X-100 and 0.05% Protease Inhibitor Cocktail) with 30 min incubation on ice. Samples were boiled and separated in 10% SDS-PAGE, transferred on to Protran^®^ Premium nitrocellulose membrane (Amersham 10600008) and blocked in blocking buffer (PBS, 0.1% Triton, 5% milk powder) overnight at 4°C. Blots were washed and incubated for 1 h with primary antibody [rat anti-HA tag (1:230, Roche 11867423001) and mouse anti-Hsp70 (1:1000) in dilution buffer (PBS, 0.1% Triton, 1% milk powder)]. After incubations, membranes were washed and incubated for 1 h with secondary antibody [IRDye^®^ 680LT Goat anti-Rat IgG Secondary Antibody (1:10000, Li-Cor 926-68029) and IRDye^®^ 800CW Goat-anti-Mouse Antibody (1:10000, Li-Cor 925-32210)]. Bands were visualized using the Li-Cor Odyssey Infrared Scanner.

### Immunogenicity prediction and peptide selection

Immunogenicity of PvSDP1 was predicted using online available tools (http://tools.iedb.org/bcell/) based in previously published algorithms (Kolaskar and Tongaonkar, 1990; Jespersen et al., 2017) (**Figures EV2A-B**). Matching regions were detected and those with higher score were used to choose a peptide for synthesis: SFTVIEKKHLSKNFKKC. Synthesized peptide was characterized by an A-HPLC with a Column Luna C18 (4.6Å~ 50mm, 3um; Phenomenex), a Gradient: Linear B (0.036% TFA in MeCN) into A (0.045% TFA in H2O) over 15 min with a flow rate of 1 ml/min and detection at 220 nm. Peptide was 97% pure and was resuspended in ultrapure H_2_O (MiliQ water), aliquoted and stored in −20°C until use. The peptide was coupled Keyhole limpet hemocyanin (KLH) at the Department of Experimental and Health Sciences – Peptide synthesis Facility of Universitat Pompeu Fabra at Centre for Genomic Regulation (Barcelona-Spain) according to their own standard operational procedures.

### Generation of polyclonal antibodies against PvSDP1 and antibody testing through ELISA

Six mice (3 female and 3 male) were immunized following protocols approved by the Catalan Government (CEEA-IGTP 19-031-HPO). Briefly, 50 μg of the synthetic peptide linked with KLH were used for individual subcutaneous injections. 0.5 ml of whole blood was extracted before immunization. Two distinct boosting injections were performed at day 21 and 42. Finally, at day 49 mice were exsanguinated and 1 ml of blood was collected from each mouse (**Figure EV2D**). Sera from individual mice was recovered after let it sit blood overnight at 4°C.

Sera collected from the terminal bleed (Day 49) from individual mice were tested for recognition of synthetic peptides of PvSDP1 by ELISA. Flat-bottom 96 well, microtitre ELISA plates were coated with PvSDP1 peptide (100 ng/well) in carbonate-bicarbonate buffer at 4°C overnight. Plates were washed three times using PBS with 0.2% Tween 20 and blocked with 5% skimmed milk in PBS for 2 h at RT. Antigen coated wells were incubated with sera samples (1:100, 1:200 or 1:1000) for 1 h at room temperature, washed and incubated with secondary antibody Goat anti-mouse IgG (Fc): HRP (Sigma A8786) at 1/500 dilution. Optical density was measured at 450 nm using Varioskan Flash equipment (Thermo Scientifc) (**Figure EV2E**).

### Indirect immunofluorescence assay

Transgenic parasite slides were prepared as previously described (Bernabeu et al., 2012). Briefly, a 10-ml culture of 10% parasitaemia of mixed stages of the *P. falciparum* 3D7_PvSDP1 transgenic line was washed in PBS and fixed with 4% EM grade paraformaldehyde and 0.075% EM grade glutaraldehyde in PBS. Fixed cells were permeabilized with 0.1% Triton X-100 in PBS and blocked for 1 h at room temperature in 3% PBS-Bovine Serum Albumin (PBS-BSA). A clinical isolate collected from a *P. vivax* malaria patient in Cambodia during field surveys by Institut Pasteur du Cambodge was used for IFA. *P. vivax* parasites were collected from peripheral blood from infected individuals as described (Martinez et al., 2024). Briefly, parasites were enriched by Percoll gradient (57% Percoll in KCl buffer) as described previously (Rangel et al., 2018) and then IFA slides were prepared by depositing cells on them, air dried and fixed with acetone:methanol (90:10) for 10 min and air dried.

Slides were incubated overnight with primary antibody [rabbit anti-HA (1:50, Invitrogen 71-5500) or mouse anti-RESA (1:200, gently donated by Professor Klavs Berzins, Stockholm University, Sweden) or mouse anti-SDP1 sera (1:20) or guinea pig anti-PvMSP1-19 (1:200, homemade) or guinea pig anti-LP1 (Bernabeu et al., 2012) (1:200)] diluted in 3% PBS-BSA. After washing, 1 h incubation with secondary antibody [anti-rabbit conjugated with Alexa Fluor 488 (1:200, Invitrogen A11008) or anti-mouse conjugated with Alexa Fluor 546 (1:200, Invitrogen A-10036) or anti-guinea pig conjugated with Alexa Fluor 647 (1:200, Invitrogen A21450)] diluted in 3% PBS-BSA was performed. Nuclei were stained for 10 min with 4,6-diaminido-2-phenylindole (DAPI, 1:1000 in PBS). Then slides were mounted using ProLong™ Gold Antifade mounting media.

Confocal microscopy images were obtained using Abberior Infinity microscope (Abberior Instruments GmbH) using 405 nm, 485 nm, 561 nm excitation laser lines. The fluorescence excitation and collection was performed using 60x/1.42 oil immersion objective. All acquisition operations were controlled by Lightbox software (Abberior Instruments GmbH). Acquired images were processed using Fiji/ImageJ software (version 1.54, NIH, USA).

### Extracellular vesicles isolation, bead based assay characterization & EVs pooling.

EVs from plasma samples of *P. vivax* infected individuals (PvEVs) and healthy donors (hEVs) (**Table EV1**) were isolated by size-exclusion chromatography (SEC) as previously published (Toda et al., 2020) with some minor modifications. Briefly, 1 ml of plasma was thawed on ice and centrifuged at 2000 x *g* for 10 min at 4°C. Supernatants were loaded on top of 10 ml handmade Sepharose CL-2B columns (Sigma 17014001), pre-equilibrated with sterile PBS. Fifteen fractions of 500 μl each were collected in 1.5 ml low-protein retention tubes (Eppendorf, 525-0133) with PBS as elution buffer and kept at -80°C until use. Each individual SEC fraction from individual patients was identified by bead-based flow cytometry assessing the presence of CD9, CD63, CD81, CD71 and/or CD5L classical EV markers. Finally, EVs isolated from patient’s plasma and healthy donor controls were pooled. To do so, 25 μl of peak quantified fractions of 10 PvEVs and 10 hEVs samples were mixed independently. PvEVs and hEVs pools were generated the day of the stimulation of the hSFs to avoid freezing/thawing.

### EVs isolation *via* direct immunoaffinity capture of CD71+

120 µl of plasma from the same 10 different patients infected with *P. vivax* were pooled to obtained 1.2 ml of pooled sample. Similar procedure and pooling was done with plasma from the same healthy donors (**Table EV1**). Pooled plasmas were diluted 1:4 in cold PBS and ultracentrifuged at 120,000 x g for four hours in a TH-641 Swinging bucker rotor (Sorvall WX, Thermofisher Ultracentrifuge 15342177) to pellet total EVs. DIC was performed as previously described (Aparici-Herraiz et al., 2022). Anti-CD71 antibody (Abcam ab214039) was coupled to Magnetic beads from Dynabeads Antibody Coupling Kit (ThermoFisher 14311D). Immediately after the last wash, captured EVs (DIC EVs) were resuspended in 35 µl SB buffer (ThermoFisher 14311D). 5 µl of DIC EVs were used for characterization of EVs by Western Blotting and 30 µl were used for functional assays.

### Human spleen fibroblast culture and stimulation with Evs

hSFs were isolated from cadaveric patients from the Transplant Programme at the Hospital Germans Tries I Pujol (Toda et al., 2020). Donation of these organs for use in biomedical research received written consent from family members and was in accordance with the protocol approved by the Ethics Committee for Clinical Research of the Hospital Germans Trias I Pujol. hSFs were cultured in Dulbecco’s modified Eagle’s Medium (DMEM) supplemented with 10% fetal bovine serum (FBS) (Gibco, 16000-044) and 1% penicillin/streptomycin solution (Gibco, 15070-063) (cDMEM) at 37°C and 5% CO_2_. To assess biocompatibility of the Magnetic Dynabeads with the hSFs, 2.5 x 10^5^ hSFs were cultured for 48 h with the Magnetic Dynabeads coupled to the CD71 antibody (ThermoFisher 14311D). Also, same number of cells were cultured without the beads for comparison. After 48 h, and after trypsinization, cells were counted and viability was assessed using Neubauer Chamber and TrypanBlue counting.

For the functional binding assay, hSFs at 70-80% confluency were trypsinized and 1.5 x 10^5^ hSFs were seeded on 13ømm coverslips (Nunc 174950) in 24-well plates in cDMEM. 24h after seeding, only adhered cells were kept and 500 μl of EVs-depleted cDMEM with 50ul of previously pooled PvEVs, hEVs or PBS independently with the SEC-purified EVs or 10 μl of the CD71^+^ captured EVs with the magnetic Dynabeads was added to each well. Each stimulation was performed in technical triplicate and with biological triplicate. EVs stimuli was kept for 48 h at 37°C before performing the functional binding assay with the *P. falciparum* 3D7_PvSDP1 transgenic line.

### *P. falciparum* 3D7_PvSDP1 functional binding assay

Stimulated hSFs were put into contact with *P. falciparum* 3D7_PvSDP1 or *P. falciparum* 3D7 wild type. Cells were washed from growing medium and 500 µl of binding medium (RPMI supplemented with 10% AB serum (Sigma H4522-100ML) at 6.8 pH), added. Afterwards, 7.5 x 10^5^ mature stage parasites purified by LS-MACS (Miltenyi 130-042-401) were added to each well independently. Parasites were incubated at 37°C, 5% CO_2_ for 1 h and unbound cells were washed thoroughly 3 times with prewarmed binding medium. Samples were fixed with methanol for 1 min and stained with 10% Giemsa. The number of parasites bound to cells were count twice independently by three different researchers (AAH, MNF, CFB) in 1000 cells of each preparation using bright-field optical microscope (Nikon, Eclipse CYL) with x100/1.25 oil objective. Statistics were calculated using Two-way ANOVA with Sidak’s multiple comparisons test.

### Binding inhibition assay using anti-PvSDP1 antibody

*P. falciparum* 3D7_PvSDP1 transgenic line was incubated with sera from immunized mouse with the PvSDP1 immunogenic peptides for 1 h at 37°C at two different concentrations of the antibody (1:5 and 1:20) diluted in binding medium. After incubation, PvEVs-stimulated hSFs were put into contact with *P. falciparum* PvSDP1 and parental *P. falciparum* 3D7. As described before, parasites were incubated at 37°C, 5% CO_2_ for 1 h and unbound cells were washed thoroughly 3 times with prewarmed binding medium. Number of parasites bound to cells were counted as described previously by three different researchers (AAH, MNF, CFB).

### Single-cell RNA

To further investigate the impact of EVs on hSFs, we conducted 10x single-cell RNA sequencing. A total of 1.5 x 10^5^ hSFs were seeded in a 6-well flat-bottom plate. Once 70-80% confluence was reached, EVs-depleted DMEM was introduced to each well. Subsequently, 100 µl of PvEVs was added to two wells, while 100 µl of PBS was added to two other wells. After 30 h of stimulation, cells were trypsinized and counted using a Neubauer chamber with Trypan Blue for viability assessment. The transcriptome of both stimulated and non-stimulated hSFs were sequenced using the Next GEM Single Cell 3’ Reagents kits v3.1 (10x Genomics), following the manufacturer’s instructions. A total of 1.6 x 10^5^ hSFs, from both the stimulated and non-stimulated groups, were independently loaded onto the Chromium Controller instrument (10X Genomics) to generate single-cell gel bead-in-emulsions (GEMs).

### Single-cell RNA-Seq data processing

Sequencing results from EVs-stimulated and non-stimulated samples, generated with 10X RNAseq, were aligned and quantified against the human reference genome GRCh38 using Cell Ranger software (Version 6.1.2) with default settings. The obtained results from Cell Ranger and the count matrix were read using the Read10X function from the Seurat library (Version 4.9.9). Subsequently, standard procedures in Seurat were employed to analyze the samples, which were initially treated independently and later merged. The analysis began with quality control, removing cells with fewer than 300 genes and those with a mitochondrial RNA content exceeding 10%. Doublets were identified using the DoubletFinder library (Version 2.0.3), integrated into the Seurat pipeline, to discern doublets within each sample. Following filtering, a total of 10,338 cells in the EV-stimulated sample and 8,894 cells in the non-stimulated sample were retained.

Normalization of the samples was performed using the LogNormalize function from Seurat, ensuring global normalization to equalize the total expression of each gene across cells. Subsequently, 2,000 Highly Variable Genes (HVGs) were selected using the FindVariablesFunction. Data were scaled using ScaleData, and dimensionality reduction was conducted based on the previously calculated HVGs. This reduction was achieved using RunPCA from Seurat, considering the first 20 dimensions. To facilitate clustering, the FindNeighbors and FindClusters functions were employed, utilizing the Louvain algorithm. Visualization of clusters was accomplished using UMAP.

### Identification of differentially expressed genes

To identify genes that were differentially expressed after stimulation with plasma-derived PvEVs, the FindAllMarkers function from Seurat was employed with default settings. A non-parametric Wilcoxon test with Bonferroni correction was applied for statistical analysis. Subsequently, the DotPlot function from Seurat was used to visually represent the differentially expressed genes of interest. To further study the clusterization of the samples, Gene ontology enrichment of human proteins for all categories was done with the Database for Annotation, Visualization, and Integrated Discovery (David 6.8) (Huang, Sherman and Lempicki, 2008). Only clusters with more than 50% of treated cells were analyzed.

### Single-Cell RNAseq validation through RT and qPCR of EVs-Stimulated hSFs

1.5 x 10^5^cells were seeded in a flat-bottom 24-well plate in exosome-depleted complete Dulbecco’s Modified Eagle Medium (cDMEM), as previously described. Independently, 50 µl of PvEVs, hEVs, or PBS was added to each well. After 48 hours of stimulation, the supernatant was removed, and 1 ml TRIzol™ (Invitrogen 15596026) was added to each well. Cells were scraped and embedded in the chaotropic agent, followed by incubation for 5 min at room temperature and freezing at -80°C until RNA extraction was performed. Biological triplicates were conducted using three different sets of PvEVs and hEVs.

RNA extraction, as described earlier in this paper, was performed, resuspended in 50 µl of DNAse/RNAse-free water, and integrity was assessed using the BioAnalyzer. First-strand cDNA synthesis utilized 1 µg of total RNA and SuperScript™ IV (Invitrogen 18091050) with Oligo(dT) primers to match the poly-A tail of mRNA. Quantitative real-time PCR reactions were carried out in technical triplicate for each biological replicate using the Light Cycler 480 Roche system (Life Science). The qPCR reactions were prepared with 5 μL of TaqMan^®^ Fast Advanced Master (Thermo Fisher Scientific), 0.5 μL of each amplification primer, 2.5 μL of nuclease-free water, and 2 μL of cDNA templates.

The PCR program included UNG incubation at 50°C for 2 min, polymerase activation at 95°C for 20 s, followed by 45 cycles of denaturation at 95°C for 1 s, annealing at 60°C for 20 s, and a negative control using 2 μL of nuclease-free water instead of cDNA. Primers used (ThermoScientific) are listed below. To normalize expression levels (ΔCp), Glyceraldehyde-3-Phosphate Dehydrogenase (*gapdh*) was employed as an endogenous control. The studied genes included CD44 Molecule (Indian Blood Group) (*cd44*), Integrin Subunit Alpha 1 (*itga1*), Intercellular Adhesion Molecule 1 (*icam1*), Thrombospondin 1 (*thbs1*), Fibronectin 1 (*fn1*), Adhesion Molecule with Ig Like Domain 2 (*amigo2*), CD36 molecule (*cd36*), Fibroblast Growth Factor 8 (*fgf8*), Toll Like Receptor 4 (*tlr4*), and C-X-C Motif Chemokine Ligand 12 (*cxcl12*). Expression levels were normalized to the internal reference *gapdh* and calculated using the 2−(ΔΔCt) formula.

## Results

### Generation of a transgenic *P. falciparum* clonal line expressing PvSDP1

**Figure 1:**
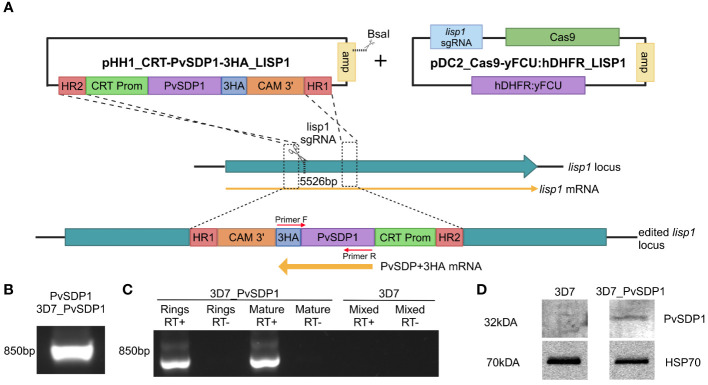
Generation and characterization of the transgenic 3D7_PvSDP1 line. **(A)** Overview of the strategy followed for the lisp1 locus editing using pDC_Cas9-yFCU:hDHFR LISP1 plasmid through CRISPR/Cas9. Scissors indicate the position targeted by the guide RNA, where Cas9 cleavage is produced. HR1 and HR2 refers to homology regions. CRT promoter is used for active transcription throughout asexual cycle. 3HA tag is used as fusion protein to enable detection and tracking of PvSDP1. Created with BioRender.com. **(B)** PCR of genomic DNA from transgenic 3D7_PvSDP1 parasites to amplify both PVX_114580 and the 3HA tag within the fusion cassette. Primer localization marked with red arrows. **(C)** RT-PCR of RNA extracted from 3D7_PvSDP1 parasite line and 3D7_WT. **(D)** Western Blot of the 3D7_PvSDP1 transgenic line. Anti-HA antibody was used for PvSDP1 protein detection, and the anti *P. falciparum* HSP70 served as the loading control.

PvSDP1 (PVX_114580) was previously described as a spleen-dependent hypothetical protein associated with clinical protection (Fernandez-Becerra et al., 2020). To further understand the function of PvSDP1, we generated transgenic *P. falciparum* 3D7 line expressing it. To do so, we modified the pHH1_DiCre_lisp1 vector (Llorà-Batlle et al., 2020) introducing the Chloroquine Resistance Transporter (CRT) promoter to ensure stable transcription of PvSDP1 through all stages of the intraerythrocytic replicative cycle (Crabb et al., 2004; Bernabeu et al., 2012) (**Supplementary Figure 1A**). The PvSDP1 construct targets the *lisp1* gene locus and is inserted at position 5523 of this *P. falciparum* gene (**Figure 1A**). *pvsdp1* was amplified from cDNA of the *P. vivax* Sal-1 strain and cloned into intermediate pHH1_CRT under the control of the *pfcrt* promoter region. A triple haemagglutinin (3HA) tag was added at the 3’ end of *pvsdp1* to facilitate its detection (**Supplementary Figure 1A**). DNA sequencing confirmed the correct sequence of the final vector.

### Characterization of *P. falciparum* 3D7_PvSDP1

CRISPR/Cas9 enabled to generate efficiently a transgenic line with similar growth pattern when compared to the parental *P. falciparum* 3D7 line (**Supplementary Figure 1B**), leaving the transgenic *P. falciparum* 3D7_PvSDP1 line marker-free and therefore sensitive to the drug WR99210 (**Supplementary Figure 1C**). Genotyping of the clonal transgenic line revealed correct integration of the *pvsdp1* gene at the *lisp1* locus detecting both the gene and the HA tag fused to it (**Figure 1B**). Efficient transcription of *pvsdp1* was detected by RT-PCR of both ring and mature parasites (**Figure 1C**). In addition, Western Blot analysis using an anti-HA antibody confirmed that the transgenic 3D7_PvSDP1 line correctly expressed the PvSDP1 protein fused to the 3HA-tag (**Figure 1D**).

### Immunofluorescence analysis reveals membrane localization of PvSDP1

**Figure 2:**
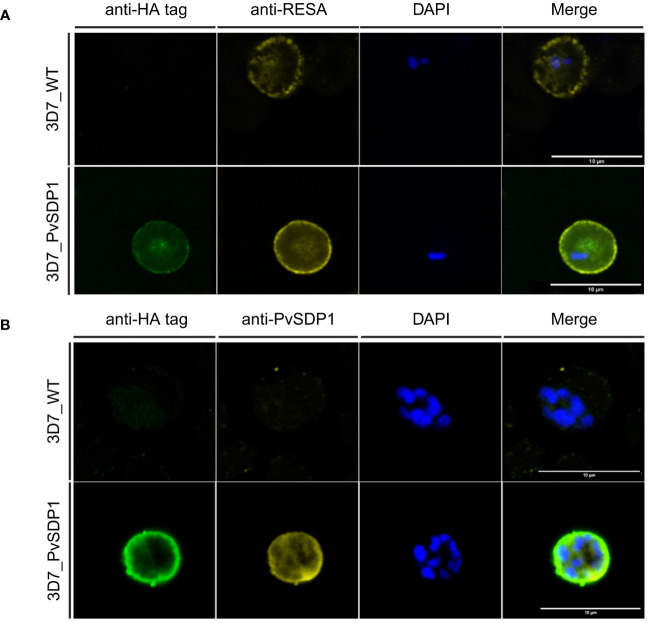
Immunofluorescence assay of 3D7_PvSDP1 transgenic line. **(A)** Rabbit anti-HA tag and mouse anti-RESA antibodies were used in combination with 3D7_PvSDP1 and 3D7 wild type (3D7_WT). In the immunofluorescence assay (IFA) images, the RESA antibody and HA-tag antibody co-localize exclusively at the membrane of the infected red blood cells (iRBC) in the transgenic 3D7_PvSDP1 line. The RESA antibody targets the membrane of 3D7_WT. **(B)** Anti-HA and anti-PvSDP1 antibodies were used in combination with 3D7_PvSDP1 and 3D7_WT. The HA-tag antibody and anti-PvSDP1 antibody co-localize at the membrane of the iRBC in 3D7_PvSDP1, with no signal detected in 3D7_WT. All scale bars represent 10 µm.

To determine the subcellular localization of the PvSDP1 protein in the *P. falciparum* transgenic line, immunofluorescence assays (IFAs) were done using antibodies against HA and the ring-infected erythrocyte surface antigen (RESA), previously used as a membrane surface marker of *P. falciparum* infected red blood cells (iRBCs) (Elizalde-Torrent et al., 2021). Although a faint signal against PvSDP1 is detected in the cytosol of transgenic 3D7_PvSDP1, likely due to the forced overexpression of the PvSDP1 gene, confocal images clearly illustrate localization of the PvSDP1 at the membrane of iRBCs. This is evidenced by the co-localization of signals from the anti-HA antibody with the anti-RESA antibody (**Figure 2A**). Finally, using anti-HA antibody in combination with a polyclonal antibody raised against PvSDP1 (**Supplementary Figure 2**), we were able to observe correct targeting of the PvSDP1 to the iRBCs membrane exclusively in the transgenic line 3D7_PvSDP1 (**Figure 2B**).

### PvSDP1 is expressed in the membrane of reticulocytes in natural infections

**Figure 3:**
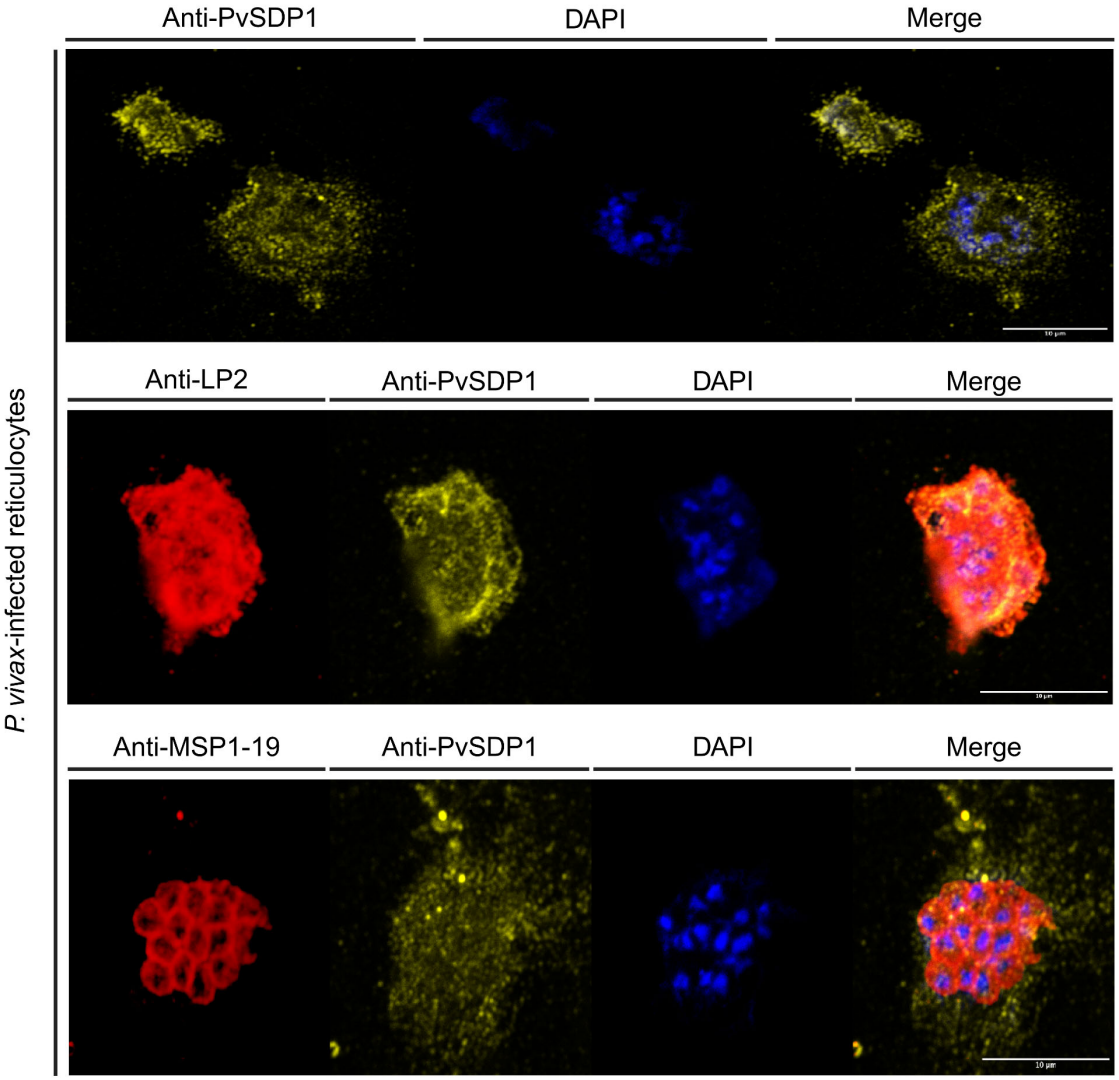
Immunofluorescence assay (IFA) on *P. vivax* field isolates. Anti-PvSDP1 antibody recognizes the SDP1 protein at the membrane of the infected reticulocyte in *P. vivax* field isolates. Anti-LP2 (targeting conserved motifs of the VIR superfamily) and anti-PvSDP1 co-localize at the membrane of the infected reticulocyte. Anti-MSP1-19 distinctly stains merozoites within the infected reticulocyte, while anti-PvSDP1 specifically targets the membrane of the infected reticulocyte. All scale bars represent 10 µm.

To confirm the subcellular localization of PvSDP1 in *P. vivax* field isolates, we used the mouse polyclonal antibody produced against PvSDP1 (**Supplementary Figure 2**) in IFA assays of *P. vivax*-infected reticulocytes obtained from a Cambodian patient. Confocal microscopy demonstrated that PvSDP1 is located at the surface of infected reticulocytes (**Figure 3**). To further confirm these results, we used the anti-PvSDP1 antibody in combination with an antibody against a long synthetic peptide (LP2) representing conserved VIR motifs, previously shown to be located at the surface of infected reticulocytes (Bernabeu et al., 2012). Co-localization of both anti-LP2 and anti-PvSDP1 antibodies is over 80% confirming membrane localization of the PvSDP1 in *P. vivax* field isolates. The anti-PvSDP1 antibody was also used in combination with an anti-PvMSP1-19 antibody displaying the typical “grape-like” shape surrounding the merozoites surface (**Figure 3**). Altogether, these results confirmed that PvSDP1 is located at the membrane of infected reticulocytes.

### Cytoadherence of *P. falciparum* 3D7_PvSDP1 to human spleen fibroblasts

**Figure 4:**
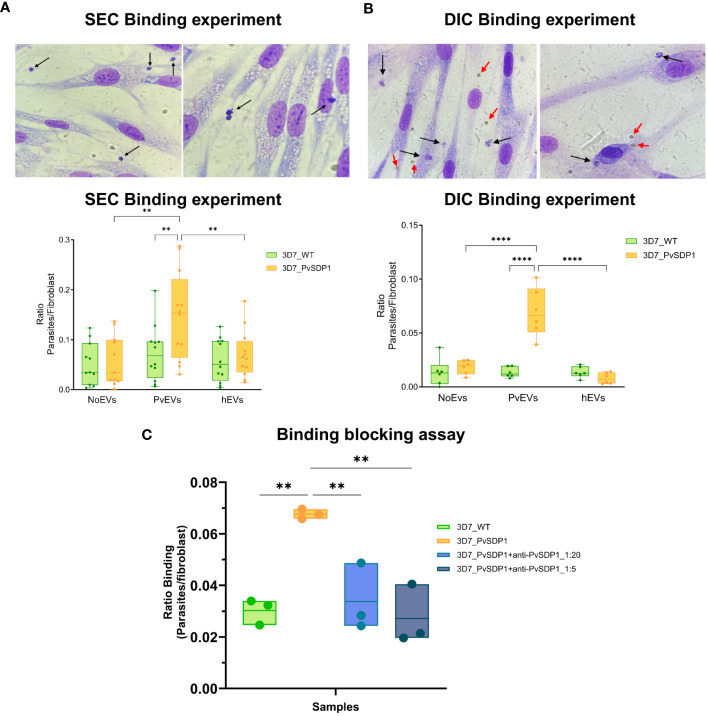
Binding of 3D7_PvSDP1 Transgenic Line to Human Spleen Fibroblasts (hSFs). **(A)** In the upper panel, Representative pictures illustrating binding of 3D7_PvSDP1 to hSFs after stimulation with SEC-purified PvEVs. Black arrows indicate parasites attached to hSFs. In Lower panel, the quantification of binding reveals a significantly increased binding capacity of the 3D7_PvSDP1 transgenic line to hSFs when cells have been stimulated with PvEVs (Two-Way ANOVA Sidak’s multiple comparisons test, **p<0.0021). **(B)** Upper panel are representative pictures depicting the binding of 3D7_PvSDP1 to hSFs after stimulation with DIC-purified PvEVs. Black arrows point to the parasite, and red arrows indicate the presence of magnetic Dynabeads. Lower panel shows the quantification of DIC binding, demonstrating an increased binding capacity of 3D7_PvSDP1 to hSFs when treated with PvEVs compared to hEVs and control PBS (Two-Way ANOVA Sidak’s Multiple Comparison test, ****p<0.0001). **(C)** Binding blocking assay. After a 1-hour incubation of parasites with two different dilutions of the anti-PvSDP1 antibody (1:5 and 1:20), the binding capacity of transgenic parasites to stimulated PvEVs in hSFs is significantly reduced by the action of the anti-PvSDP1 antibody (One-way ANOVA Tukey’s multiple comparison test, **p<0.002).

It was previously shown that EVs isolated from *P. vivax* individuals increased the binding capacity to hSFs (Toda et al., 2020). For functional assays and due to the low peripheral blood volume withdrawn from patients during acute attacks, we purified plasma-derived EVs using Size Exclusion Chromatography (SEC) and pooled fractions 7, 8 and 9 from ten patients (PvEVs). As controls, we also made a single pool of the same SEC fractions from circulating EVs obtained from ten healthy donors (hEVs). SEC EVs were then characterized by Bead Based Assay (BBA), Nanoparticle Tracking Analysis (NTA) and Stimulated emission depletion (STED) microscopy (**Supplementary Figures 3A–D**). hSFs were stimulated with PvEVs and hEVs independently for 48h. Subsequently, EV-stimulated hSFs were incubated for 1 hour with the 3D7_PvSDP1 transgenic line as well as the parental 3D7_WT line, followed by thorough washing to remove unbound parasites to the cells. Binding was assessed via light microscopy (**Supplementary Figure 4A**). Ratios between hSFs and parasites were calculated, and statistics was performed using the Two-Way ANOVA. Binding of *P. falciparum* 3D7_PvSDP1 was significantly increased when hSFs were stimulated with PvEVs, when compared with wild type parasites (Sidak’s multiple comparisons test *p=0061)*. Interestingly, this effect was only seen when the hSFs have been stimulated with PvEVs but not with hEVs or control PBS (**Figure 4A**).

### Immunocaptured EVs increase binding to hSFs

We had previously shown that CD71^+^-EVs increased the signal of parasite proteins detection associated with circulating EVs from patients (Aparici-Herraiz et al., 2022). CD71^+^-EVs from patients’ plasma and healthy donors were purified by Direct Immunocapture (DIC) using magnetic Dynabeads (ThermoFisher) and characterized by western blot (**Supplementary Figure 5A**). Beads coupled to CD71 antibodies were directly added to hSFs and cell viability assessed after 48h. There were no changes in growth rates or cell viability in hSFs that have been in contact with the Dynabeads when compared with control non-stimulated cells (**Supplementary Figures 5B, C**). Next, stimulation of hSFs with CD71^+^-EVs was done and functional binding assays was performed. To compare binding of the transgenic *P. falciparum* 3D7_PvSDP1, stimulation of hSFs was also performed with CD71^+^-hEVs and Dynabeads with the CD71 antibody alone. 3D7_PvSDP1 transgenic parasites showed a significant increased binding capacity to hSFs when stimulated with CD71^+^-PvEVs compared to the parental 3D7_WT (Sidak’s multiple comparison test *p<0.0001*).

Interestingly, there was a significant increase in the binding capacity of the 3D7_PvSDP1 to hSFs previously stimulated with EVs from infection when comparing to healthy EVs or control Dynabeads (Sidak’s multiple comparison test, p < 0.0001) (**Figure 4B**). Altogether, DIC immunocaptured CD71^+^-EVs increased the statistical significance of the binding capacity of the transgenic line to hSFs, in comparison to SEC-purified EVs (**Figure 4A**). Of note, despite thoroughly washing, Dynabeads were still observed at the surface of hSFs, yet no parasites were observed adhering directly to the magnetic beads (**Figure 4B**, upper panel).

### Anti-PvSDP1 polyclonal antibodies block binding of the transgenic *P. falciparum* 3D7_PvSDP1 line to hSF

To confirm that the increase binding capacity of the transgenic line to hSF was mediated by the PvSDP1 protein, polyclonal antibodies generated against the protein were used in functional binding-inhibition assay. After incubation of the parasites with the antibody, parasites were put into contact with PvEVs-stimulated hSF for 1h as described previously. After thorough washes, binding of the parasites was quantified as previously described (**Supplementary Figure 4B**). Interestingly, after the incubation with anti-PvSDP1 sera, the transgenic 3D7_PvSDP1 parasites exhibit a reduction in the binding capacity to hSFs when compared to non-blocked 3D7_PvSDP1 parasites (One-way ANOVA *p=0.0028 in 1:5 dilution and p=0.0082 in 1:20 dilution)* (**Figure 4C**).

### Single-cell RNAseq showed increased adhesin expression in PvEVs-treated hSFs

**Figure 5:**
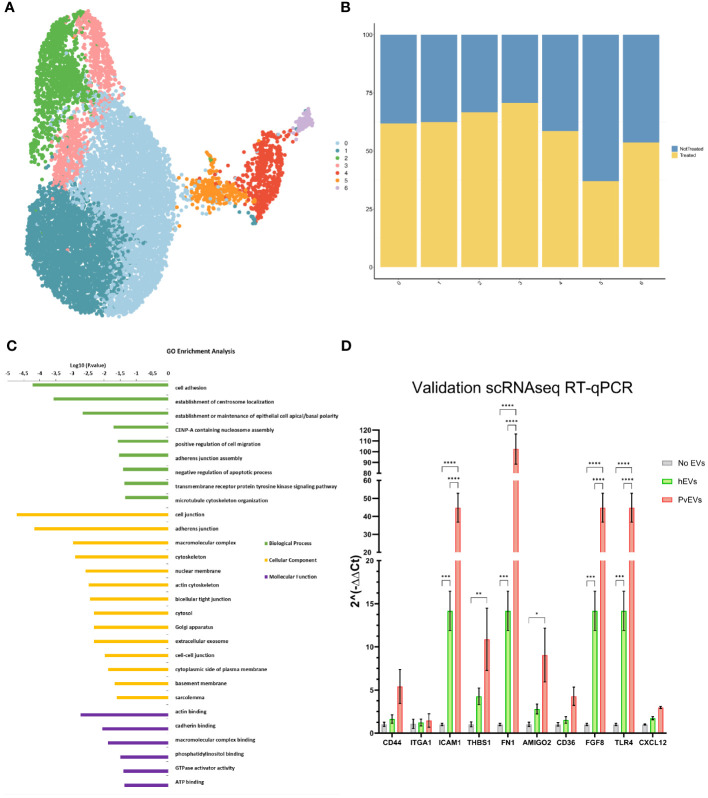
Single-Cell RNAseq Analysis. **(A)** UMAP Clusterization of the sequenced hSFs. **(B)** Bar plot illustrating the distribution of cells between not treated and PvEVs treated groups among the obtained clusters. **(C)** Gene Ontology (GO) analysis of Cluster 1. The DAVID Bioinformatic tool was employed for GO enrichment analysis. **(D)** RT-qPCR validation of selected adhesins. hSFs were stimulated with either PvEVs or hEVs. The results indicate upregulation of certain genes after stimulation with PvEVs and hEVs. The basal expression level in the absence of EVs was calculated individually for each gene (Two-way ANOVA Bonferroni’s multiple comparisons test, *p<0.0332, **p<0.0021, ***p<0.0002, ****p<0.0001).

To further investigate the effect of PvEVs over hSFs whole transcriptomic analysis was done by 10X Single-Cell RNAseq. Cells were stimulated for 48h with plasma-derived EVs followed by 10X Gel Beads-in-emulsion (GEM) preparation and amplification. In parallel, untreated hSFs were also incubated for 48h and also used for 10X GEM preparation. Following clusterization of stimulated and non-stimulated cells (**Figure 5A**), we were able to study the distribution of treated and not-treated cells in each fibroblast cluster (**Figure 5B**). To gain insight into the genes that define this cluster, a GO enrichment analysis of individual cluster with more than 50% of treated cells was performed. Of note, cell adhesion, cellular compartments related to cellular junction, exosomes and other membrane-related terms and biological processes relevant to binding, were largely enriched in Cluster 1 (**Figure 5C**). To confirm the increase expression of cell adhesion genes, we used RT-qPCR to check transcription levels of selected adhesins genes, all included in the cell adhesion GO term. CD44 (Dalimot et al., 2022), CD36 (Cabrera et al., 2014) and ICAM1 (Toda et al., 2020) have been previously reported to play an important role in malaria pathophysiology. Here, we were able to observe how after stimulation with EVs coming from infected individuals, hSFs exhibit a significant increased expression of adhesins like *fn1, amigo2, icam1, thbs1 and tlr4* (Two-way ANOVA test). Also, we observed increased expression of *cd44* and *cd36* although not being statistically significant. hEVs also produced an increase in the expression levels of the assessed genes when compared to control PBS stimulation, however, this effect is significantly smaller than PvEVs (**Figure 5D**).

## Discussion

The presence of asymptomatic infections of *Plasmodium vivax* is a major challenge towards malaria elimination. In recent years, the spleen has emerged as a key player of such infections (Siqueira et al., 2012) where more than 95% of the total parasite biomass is found in this reticulocyte-rich organ (Kho et al., 2021b, 2021a). Here, we have characterized a hypothetical *P. vivax* spleen-dependent gene (PVX_114580) (Fernandez-Becerra et al., 2020), proposed to be named *P. vivax* spleen dependent protein 1 (PvSDP1), and shown that EVs from natural infections facilitate its binding to hSFs; thus, revealing new insights into intrasplenic infections in this species.

Implementation of CRISPR/Cas9 in malaria research has enabled highly targeted and precise modifications of the malaria parasite’s genome (Ghorbal et al., 2014; Adjalley and Lee, 2022). Advances in studying *P. vivax* genes using this technology enabled to study the role of the CSP protein in *P. vivax* sporozoite formation and infectiveness (Marin-Mogollon et al., 2018). We used CRISPR/Cas9 editing to knock-in PVX_114580 into a non-essential gene for asexual stages of *P. falciparum*, the *lisp1* (Llorà-Batlle et al., 2020), under the control of the CRT promoter for constitutive expression during asexual blood stages (Bernabeu et al., 2012). We opted against substituting the *P. falciparum* ortholog (PF3D7_0629600) due to the limited homology with only a 51% identity conservation between them. Also, transcription of the *P. falciparum* 3D7 ortholog through intraerythrocytic cycle is relatively low when compared to more abundant transcripts like *msp1* (PF3D7_0930300) or house-keeping genes like *gapdh* (PF3D7_1462800) (Otto et al., 2010; Chappell et al., 2020). Results corroborated that PVX_114580 is expressed in asexual blood stages and that PvSDP1 is located at the surface of infected red blood cells (**Figures 1**, **2**).

Surface expression of PvSDP1 allowed testing this protein as a ligand in interactions with hSFs as previously reported for another *P. vivax* variant surface protein (Fernandez-Becerra et al., 2020). Moreover, as such interactions had been shown to be facilitated by EVs from natural infections (Toda et al., 2020), we used EVs isolated by SEC in binding experiments and observed an increase binding capacity of the transgenic parasite line expressing the PvSDP1 protein (**Figure 4A**). Of importance, for what we believe is the first time, we also used in these assays circulating EVs directly immunocaptured by CD71-antibodies as functional stimuli and showed an increase in the significance of the binding capacity of the transgenic 3D7_PvSDP1 to hSFs when compared to EVs isolated by SEC (**Figure 4B**). These results are likely due to the increased signal of parasite proteins associated with circulating EVS detected by proteomic analysis (Aparici-Herraiz et al., 2022). Moreover, these data suggest the use of directly immunocaptured EVs from human plasma in functional assays, thus facilitating studies of their physiological role.

PvSDP1 was initially reported as a hypothetical *P. vivax* gene (PVX_114580) with true orthologues in other malaria species (Carlton et al., 2008) and later shown to be dependent on an intact spleen for expression (Fernandez-Becerra et al., 2020). It exhibits the PEXEL exportation motif (**Supplementary Figure 1D**) (Pick et al., 2011) which is in accordance with its membrane localization when is heterologously expressed in the *P. falciparum* 3D7 strain, as confirmed by confocal imaging. This localization is further supported by its co-localization with a RESA antigen from *P. falciparum*, previously identified as a membrane-associated protein (Elizalde-Torrent et al., 2021). Noticeably, polyclonal antibodies raised against a synthetic peptide from the immunogenic region of the protein blocked the binding of PVSDP1 to hSFs after EV uptake, confirming unequivocally its cellular localization on the membrane surface of iRBCs. Moreover, we were able to confirm the surface localization of PvSDP1 in infected reticulocytes from natural infections through co-localization experiments using anti-LP2 antibody. It is worth noting, that the punctate localization pattern observed in infected reticulocytes may be attributed to the different fixation method employed, based on methanol: acetone. Due to the importance of intrasplenic infections in chronic asymptomatic infections in malaria, further studies of PVSDP1 in vaccine development are warranted.

Single-cell RNA analysis area has enabled the identification of signaling mechanisms in *P. vivax* (Sà et al., 2020; Ruberto et al., 2022). To get further insights into the EVs-induced interactions with spleen fibroblasts during *P. vivax* infections, we performed single-cell RNA analysis of hSFs after uptake of EVs from *P. vivax* patients. GO enrichment analysis revealed an over-representation of terms such as cell adhesion, cellular components related to the cell junction and adherence junctions, among others, in Cluster 1. Indeed, expression levels of certain adhesins (**Figure 5D**) like *cd36* (Cabrera et al., 2014; Nguyen et al., 2023), *icam1* (Toda et al., 2020; Gill et al., 2023), *thbs1*, *cd44* (Egan, 2018) or *thbs1* (Kanchan et al., 2015) and genes like *fgf8* (Martin-Jaular et al., 2011) were validated by RT-qPCR and is in accordance with what was previously published (Bernabeu et al., 2012; Toda et al., 2020). Together, these data strongly suggest that EVs from natural infections interact with hSFs increasing the expression of cell adhesins, thus facilitating binding of *P. vivax*-infected reticulocytes and formation of intrasplenic niches.

We are conscious of some limitations of this work: (i) functional studies of PvSDP1 using heterologous expression in *P. falciparum*, might not reveal is solely function in natural infections; (ii) the heterogeneity of human plasma EVs is always a confounding factor; yet, the use of immunocaptured CD71^+^-EVs increases the specificity of reticulocyte-derived EVs; (iii) Single-cell RNA experiments were limited to hSFs requiring future experiments with other spleen cells.

In summary, our research offers insights into how *P. vivax* utilizes circulating EVs to communicate with the human spleen, thereby facilitating the formation of cryptic intrasplenic infections. Additionally, our findings suggest that parasite genes whose expression relies on an intact spleen may serve as ligands for cell adhesins expressed in hSFs. A critical question that remains unanswered is the mechanism underlying the selectivity of EVs to interact with specific cells within this complex organ. Further transcriptional studies of the human spleen using *in vivo* and *in vitro* models as well as from spleen ruptures during asymptomatic infections are necessary for a comprehensive understanding of extracellular vesicle-mediated intercellular communication and intrasplenic infections.

## Data availability statement

The datasets presented in this study can be found in online repositories. The names of the repository/repositories and accession number(s) can be found below: https://www.ncbi.nlm.nih.gov/geo/, GSE255891.

## Ethics statement

The studies involving humans were approved by Comite de Etica de la Universidad de Cordoba, Monteria, Colombia and Ethics Committee Hospital Germans Tries I Pujol, Badalona (Spain). The studies were conducted in accordance with the local legislation and institutional requirements. The participants provided their written informed consent to participate in this study. The animal study was approved by Comitè Ètic d’Experimentació Animal de l’Institut de Recerca Germans Trias i Pujol. The study was conducted in accordance with the local legislation and institutional requirements.

## Author contributions

AA-H: Writing – review & editing, Writing – original draft, Visualization, Validation, Resources, Methodology, Investigation, Formal analysis. MN-F: Writing – review & editing, Validation, Resources, Methodology, Investigation. AL: Writing – original draft, Visualization, Validation, Software, Methodology, Formal analysis, Data curation. IA-H: Writing – review & editing, Resources, Methodology, Investigation. ET-F: Writing – review & editing, Resources, Methodology. OL-B: Resources, Methodology, Writing – review & editing. AO: Resources, Writing – review & editing. MY: Resources, Writing – review & editing. MG: Methodology, Writing – review & editing. ME: Resources, Writing – review & editing. JP: Resources, Writing – review & editing. AC: Resources, Methodology, Writing – review & editing. HP: Writing – review & editing, Writing – original draft, Supervision, Resources, Project administration, Methodology, Funding acquisition, Formal analysis, Conceptualization. CF-B: Writing – review & editing, Writing – original draft, Validation, Supervision, Resources, Project administration, Methodology, Investigation, Funding acquisition, Formal analysis, Conceptualization.
