# Supplementary material for: Plasmodium vivax spleen-dependent protein 1 and its role in extracellular vesicles-mediated intrasplenic infections
Source: Front Cell Infect Microbiol. 2024 May 17;14:1408451. doi: 10.3389/fcimb.2024.1408451 (PMC11140020; doi:10.3389/fcimb.2024.1408451)
Supplement: Supplementary file 1 [file DataSheet_1.pdf]

SUPPLEMENTARY DATA FILE

Table of Contents

Supplementary Figures 2

Supplementary Figure 1. Characterization of 3D7_PvSDP1 Transgenic Line and Plasmid Map. 2

Supplementary Figure 2. Generation of polyclonal antibodies against PvSDP1. 3

Supplementary Figure 3. Characterization of SEC EVs used for functional assays. 4

Supplementary Figure 4. Schematic representation of functional assays. 5

Supplementary Figure 5. Characterization of DIC-captured EVs and investigation of the effect of Dynabeads over hSFs. 6

Supplementary Table 7

Supplementary Table 1. Information of *P. vivax* infected individuals and healthy donor controls samples used for functional assay 7

1 Supplementary Figures

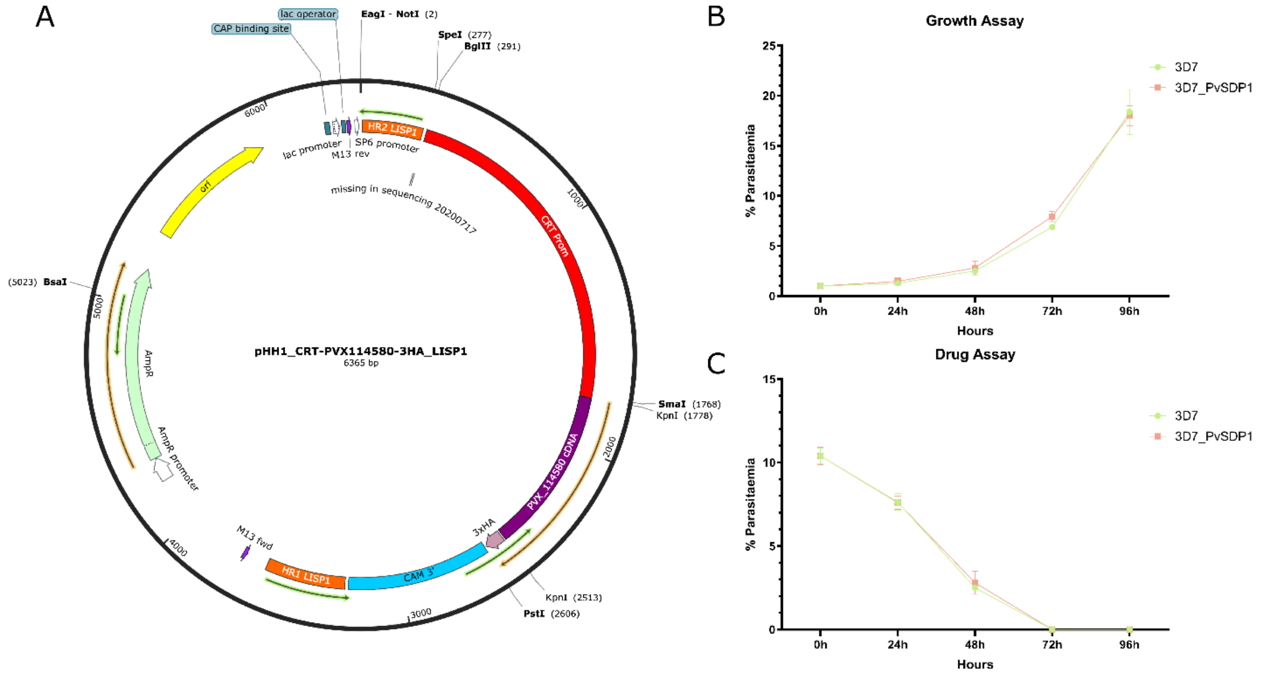

D

>PVX_114580
MKSILGHFRILSIPVEDNPLTTINLLVANCKDISEHDNAQSDALCAAALDNIVGEEDLKSFFSNFGKINSFTVIEKKHLS
KNFKKFAYCAYVTYRENNVVEIILQYSIHVDDYFKSSVQSPYFINLEKKKFAYYIKKYYENYYDLYNERKIIINEISKIN
KENNKGKKKLGLVDEDGFTVVQRVADKPEFINSVFVPANDRFSYLKKERKMKIHENFYLFQKKESIRNASELLAKKKNKQ
KKF

1.1 Supplementary Figure 1. Characterization of 3D7_PvSDP1 Transgenic Line and Plasmid Map.

(A) Plasmid Map containing PVX_114580.

(B) Growth assay comparing parental and transgenic 3D7_PvSDP1 line.

(C) Drug (WR99210) sensitivity assay comparing parental and transgenic 3D7_PvSDP1 line.

(D) Aminoacidic sequence of the SDP1 protein. In yellow, the exportation PEXEL-motif highlighting conserved sequences of this motif (Pick *et al*, 2011).

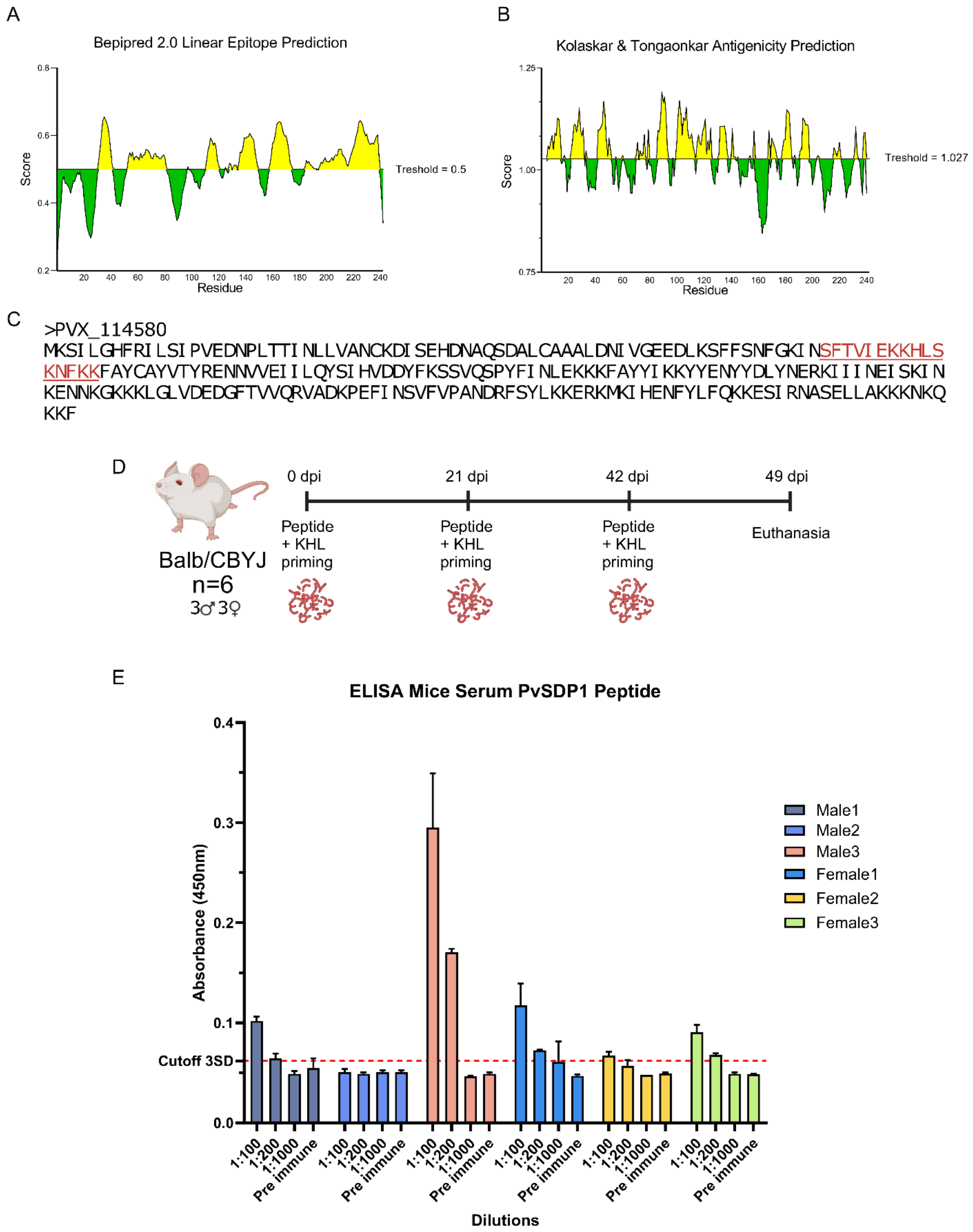

1.2 Supplementary Figure 2. Generation of polyclonal antibodies against PvSDP1.

(A) Bepipred linear epitope prediction results of the protein sequence of PvSDP1. Immunogenicity of PvSDP1 was predicted using online available tools (http://tools.iedb.org/bcell/) (Kolaskar and

Tongaonkar, 1990; Jespersen et al., 2017), Matching regions were detected and those with higher score were used to choose a peptide for synthesis: SFTVIEKKHLSKNFKKC

(B) Antigenicity Kolaskar-Tongaonkar prediction algorithm of the protein sequence of PvSDP1.

(C) Protein sequence of PvSDP1. Note in red underline is the peptide selected for the immunization of mice.

(D) Immunization strategy of Balb/CBYJ mice using immunogenic synthetic peptide from PvSDP1

(E) ELISA results from mice sera obtained after immunization with PvSDP1 peptides.

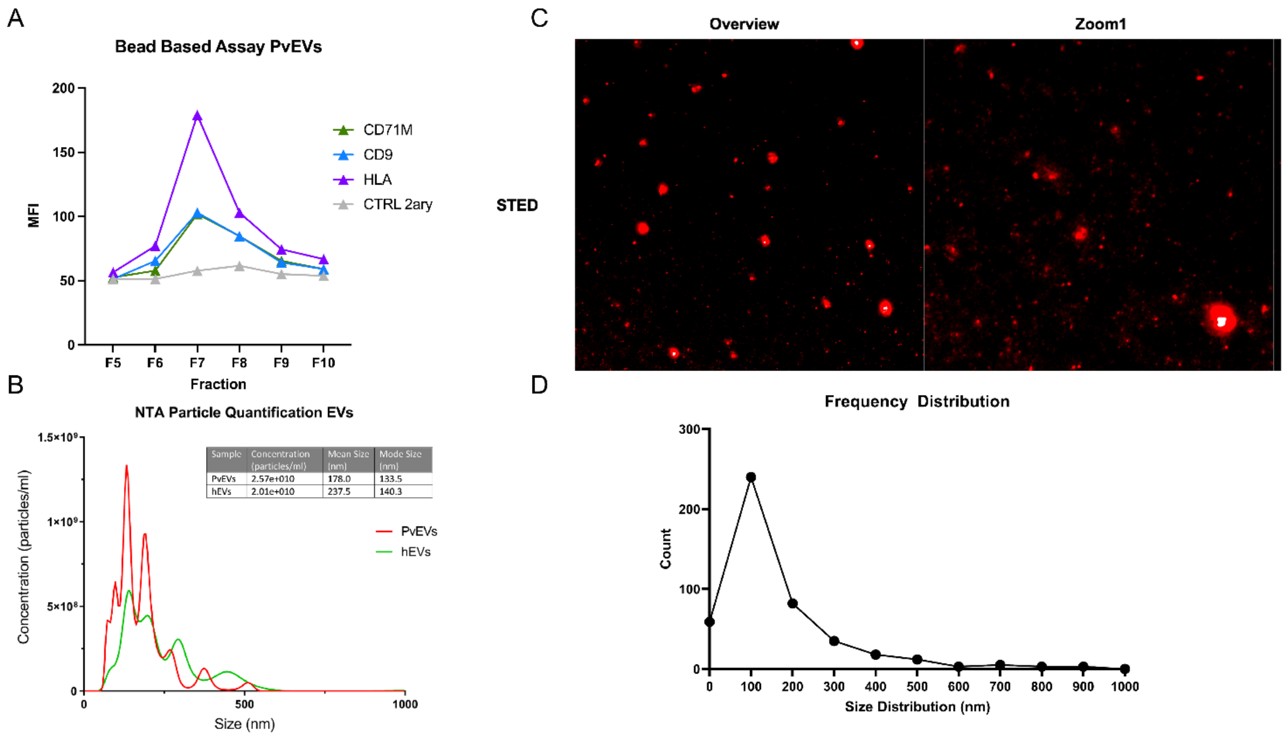

1.3 Supplementary Figure 3. Characterization of SEC EVs used for functional assays.

(A) Representative Bead-based Assay of PvEVs where CD9, HLA, and CD71 served as EV markers in all PvEVs or hEVs SEC.

(B) NTA Particle Quantification of EVs.

(C) Images corresponding to Super Resolution STED Microscopy where DPPE membrane staining is utilized to stain PvEVs.

(D) Automated quantification of the Size Distribution of PvEVs Stained with DPPE and Imaged in STED Mode.

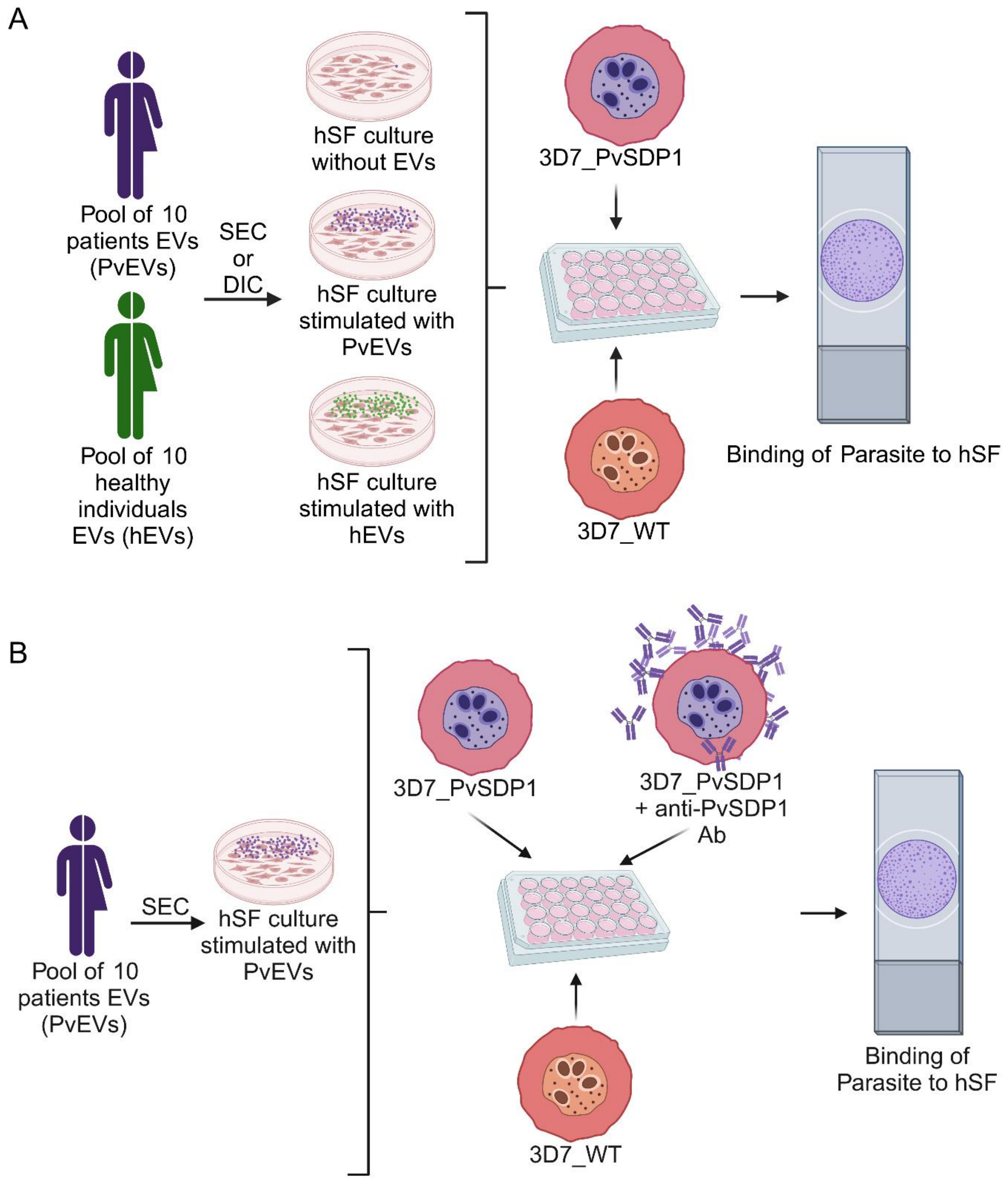

1.4 Supplementary Figure 4. Schematic representation of functional assays.

(A) Binding Experiment schematic representation.

(B) Binding inhibition assay using anti-PvSDP1 antibody.

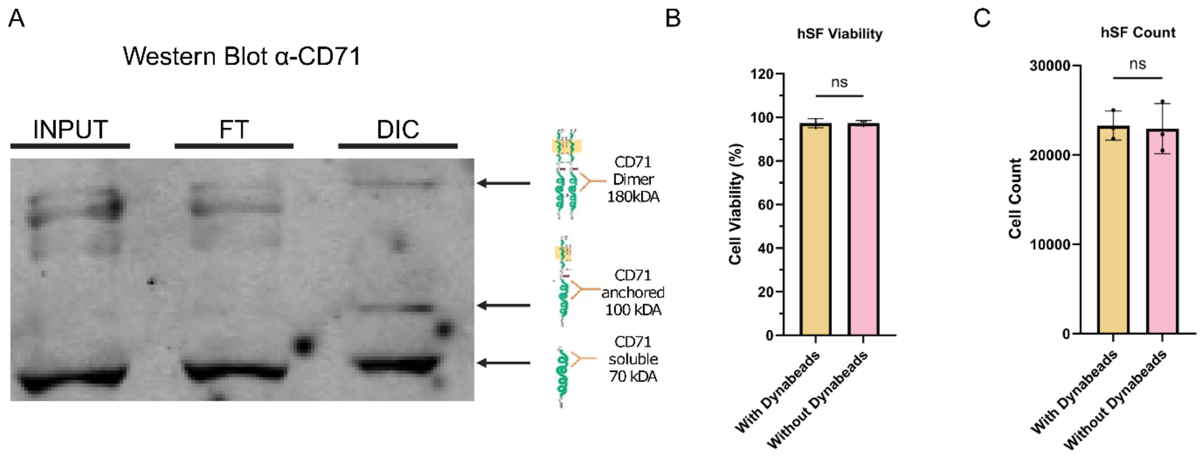

1.5 Supplementary Figure 5. Characterization of DIC-captured EVs and investigation of the effect of Dynabeads over hSFs.

(A) WB of Direct-Immunocaptured CD71+ EVs. In the Direct Immunocapture (DIC) fraction, there is an enrichment in the anchored CD71 form and CD71 dimer, both associated with EVs.

(B) hSFs viability test after incubation for 48 h with magnetic Dynabeads coupled with anti-CD71 antibody.

(C) hSFs count by microscopy using Neubauer Chamber after incubation for 48 h with magnetic Dynabeads coupled with anti-CD71 antibody.

2 Supplementary Table

Patient code	Collection Date	Residence time in malaria endemic area	City	State	Country	Age (years)	Parasitaemia (parasites/µl)	Number of previous malaria infections	Gender	Pregnant
B8-04	22/11/2018	130 months	Tierralta	Cordoba	Colombia	31	5200	3	M	
B8-09	28/11/2018	9 months	Tierralta	Cordoba	Colombia	0,75	2100	0	M	
B8-10	28/11/2018	60 months	Tierralta	Cordoba	Colombia	10	2000	0	M	
B8-11	28/11/2018	48 months	Tierralta	Cordoba	Colombia	40	2000	1	M	
B8-12	28/11/2018	48 months	Tierralta	Cordoba	Colombia	4	19310	0	M	
B8-13	29/11/2018	276 months	Tierralta	Cordoba	Colombia	23	18600	2	F	NO
B8-14	30/11/2018	156 months	Tierralta	Cordoba	Colombia	13	2901	3	M	
B8-16	30/11/2018	600 months	Tierralta	Cordoba	Colombia	64	2325	1	F	NO
B8-27	24/05/2019	N/A	Tierralta	Cordoba	Colombia	39	4219	N/A	F	NO
B8-33	06/06/2019	N/A	Tierralta	Cordoba	Colombia	20	2610	N/A	M	
HD-04	11/01/2019	0	Badalona	Barcelona	Spain	34	-	0	M	
HD-05	24/01/2019	0	Badalona	Barcelona	Spain	23	-	0	M	
HD-07	24/01/2019	0	Badalona	Barcelona	Spain	24	-	0	F	NO
HD-09	08/03/2019	0	Badalona	Barcelona	Spain	37	-	0	F	
HD-11	08/03/2019	0	Badalona	Barcelona	Spain	33	-	0	M	
HD-14	18/06/2019	0	Badalona	Barcelona	Spain	25	-	0	F	NO
HD-15	18/06/2019	0	Badalona	Barcelona	Spain	25	-	0	M	
HD-20	18/04/2023	0	Badalona	Barcelona	Spain	24	-	0	F	NO
HD-22	18/04/2023	0	Badalona	Barcelona	Spain	29	-	0	F	NO
HD-23	18/04/2023	0	Badalona	Barcelona	Spain	48	-	0	M	

N/A: Information not available

2.1 Supplementary Table 1. Information of *P. vivax* infected individuals and healthy donor controls samples used for functional assay
